# Generative AI-driven synthetic media risks in digital health: implications for telemedicine and teledentistry

**DOI:** 10.3389/fpubh.2026.1781216

**Published:** 2026-02-19

**Authors:** Karol Jędrasiak, Julia Bijoch

**Affiliations:** 1 WSB University, Dabrowa Gornicza, Poland; 2 Collegium Medicum - Faculty of Medicine, WSB University, Dabrowa Gornicza, Poland

**Keywords:** audiovisual integrity, deepfake detection, digital health security, explainable artificial intelligence, generative artificial intelligence, multimodal coherence, telemedicine

## Abstract

Advances in diffusion-based and neural rendering architectures have enabled the creation of synthetic audiovisual content that closely replicates natural facial dynamics, speech production, and environmental context. These developments pose a growing risk to clinical medicine and dentistry, where authentic audiovisual data support remote clinical assessment, communication, and medico-legal documentation. This study introduces an interpretable multimodal framework for deepfake detection that integrates visual, acoustic, and cross-modal coherence features, with decision thresholds derived exclusively from authentic recordings to ensure transparency and forensic accountability. Using the DeepFake RealWorld dataset comprising 46,371 audiovisual clips, including 77% with audio, we evaluated 47 descriptors across optical, bioacoustic, and synchronization domains. Clinical relevance was evaluated through simulated dental teleconsultations. Cross-modal metrics, particularly lip-speech synchrony (Δp = 0.21–0.22), phoneme-viseme alignment (Δp = 0.21), a widely used audio visual consistency cue in multimodal deepfake detection, identity coherence (Δp = 0.19), and scene-audio semantic consistency (Δp = 0.18) demonstrated the strongest discriminatory performance, with prevalence ratios of up to 2.7. Acoustic markers, including reduced jitter, shimmer, and shortened reverberation time (RT60; 0.12 s in synthetic vs. 0.28 s in real recordings), provided additional robustness. The framework maintained performance degradation below 15% under platform-scale compression and recapture artifacts. Additionally, the proposed framework was benchmarked against a standard open-source texture-oriented baseline detector based on the Xception architecture, with clip-level ROC AUC and balanced accuracy reported on the original clips and under the same platform transformations used in the robustness analysis. Simulated dental teleconsultations revealed that manipulated recordings introduce inconsistencies in mandibular motion, prosody-related facial dynamics, and ambient acoustic plausibility (mean Δp = 0.18; PR = 2.3), confirming the clinical relevance of multimodal coherence analysis. These results position coherence-based detection as a reliable, transparent, and domain-appropriate approach for safeguarding audiovisual integrity in remote dentistry, medicine, and related digital health applications.

## Introduction

Recent advances in generative artificial intelligence, particularly in diffusion-based architectures and neural rendering, have transformed the synthesis of audiovisual content, enabling the creation of highly realistic simulated facial motion and speech. The removal of visual and acoustic artifacts that previously facilitated forensic identification has complicated the authentication of digital recordings in high-stakes sectors, including healthcare (1, 2). These developments pose significant challenges for medical environments in which the integrity of audiovisual data is critical for diagnostic reliability, clinical communication and medico-legal documentation (3–5). The widespread adoption of telemedicine during the COVID-19 pandemic further expanded the role of remote consultations, simultaneously increasing exposure to manipulation threats that undermine digital trust in healthcare systems (6).

Within this evolving generative AI ecosystem, large language models increasingly act as orchestration layers in generative audiovisual systems, enabling automated script generation, semantic control, and cross-modal alignment between visual, acoustic, and contextual streams. In digital health environments, this convergence of LLM-driven coordination with diffusion-based image synthesis and neural audio rendering substantially amplifies both the scalability of synthetic media and the associated clinical risks in telemedicine and teledentistry workflows.

Beyond the audiovisual domain, recent publications have highlighted the potential vulnerability of clinical data to intentional modification across various areas of medicine and dentistry (3, 6, 7). Radiological and photographic material, as well as acoustic and biomechanical recordings, represent potential vectors of clinical data alteration. If modified, such diagnostic sources could influence treatment decisions or blur the boundary between simulation and clinical documentation. Furthermore, the possibility of falsified entries in electronic health records and the emergence of fully synthetic clinical datasets generated by AI highlight broader concerns regarding data integrity in healthcare (4). In this context, deepfaked audiovisual material should be regarded not as an isolated phenomenon, but rather as the newest expression of an already established pattern of clinical data manipulation - one that compromises diagnostic reliability, medico-legal accountability and the integrity of patient-clinician communication (2, 5). The growing accessibility of synthetic media generation tools therefore introduces a new category of risk for teledentistry and telemedicine, where authenticity and patient safety are contingent on the credibility of audiovisual material.

Traditional deepfake detectors predominantly rely on unimodal features, such as frequency domain inconsistencies or local texture-based anomalies (8). These approaches, although successful in controlled environments, exhibit substantial degradation of accuracy when exposed to real world compression pipelines, scaling artifacts, low bitrate streaming, or platform specific transformations. Several studies demonstrate performance declines exceeding 30 % for detectors evaluated outside the narrow distribution of their training data platforms (9). This vulnerability is particularly problematic for teleconsultations, where recordings are routinely subject to compression and transmission artifacts inherent to consumer devices.

In contrast, multimodal coherence features that integrate visual, acoustic, and semantic cues offer increased robustness against such transformations (10). Metrics describing synchrony between facial motion and speech, temporal consistency between phonemes and visemes, scene to audio correspondence, and the alignment of facial identity with vocal characteristics provide more stable discriminative power under realistic degradation conditions. Phoneme viseme alignment is an established theme in recent multimodal deepfake detection and has been explicitly modeled in contemporary frameworks that use phoneme temporal structure and identity dynamics, as well as audio visual self supervised representations focused on the lip region. Accordingly, we treat phoneme viseme alignment as a standard component and focus on its teledentistry oriented interpretation and calibration rather than presenting the general technique as a novel discovery (11, 12). These multimodal features align closely with human perceptual expectations and physiological mechanisms of speech production, which enhance their forensic interpretability and render them compatible with emerging explainable artificial intelligence standards (8, 13, 14).

Despite their potential, current research in multimodal coherence remains limited in scope, with few studies investigating comprehensive audiovisual descriptors across heterogeneous real-world datasets. Moreover, the application of deepfake detection in teledentistry and telemedicine has received minimal attention, leaving a critical gap in safeguarding the authenticity of patient recordings. The integration of multimodal coherence into clinical workflows could significantly enhance diagnostic reliability and strengthen medico-legal trust in teleconsultation environments (12, 15).

The present study introduces a systematically designed, explainable framework for multimodal deepfake detection tailored to clinical dentistry. Using the DeepFake RealWorld dataset containing 46,371 audiovisual clips with realistic platform specific degradations, the study identifies a set of interpretable descriptors that demonstrate robust discriminative performance and maintain stability under compression. By grounding the analysis entirely in threshold-based evaluation derived from real authentic data, the framework ensures transparency and compliance with forensic requirements. The resulting findings address a significant methodological gap and establish a clinically relevant foundation for the verification of audiovisual integrity in dental practice. To substantiate the robustness claim with an external reference, we include a direct benchmark against a widely used open source detector that primarily exploits local texture and compression related artifacts. We report standard performance metrics alongside the internal activation based measures to enable objective comparison on the same dataset and transformation conditions.

Relative to our prior conference report, the main new contribution of this manuscript is an expanded and systematically evaluated set of coherence descriptors (16). We introduce additional multimodal features that quantify audiovisual synchrony, facial motion plausibility, semantic consistency between visual content and speech, and audio scene plausibility, and we evaluate their robustness under real world platform transformations. We further refine activation thresholds derived from authentic recordings and add an external benchmark against an open source Xception baseline, reporting clip level ROC AUC and balanced accuracy on original and transformed clips.

## Materials

The study was conducted using the DeepFake RealWorld dataset, a large-scale audiovisual repository comprising 46,371 clips curated to reflect the technological and distributional conditions of contemporary synthetic media. This dataset was specifically designed to address the limitations of earlier benchmarks, which predominantly relied on controlled studio recordings or generator-dependent artifacts that no longer represent the capabilities of diffusion-based and hybrid generative models, as documented in prior benchmark studies and surveys (1, 9, 17).

The DeepFake RealWorld collection contains 4,186 in the wild deepfakes gathered from open sources, complemented by 42,185 synthetically generated or transformed clips created using state of the art generative pipelines. These include diffusion-based video models, modern face swap systems, audio driven facial reenactment tools, and multimodal generation architectures that dominate the 2025 landscape. Each clip was normalized to MP4 with H.264 encoding, nominal 30 frames per second, and audio sampled at 48 kHz to ensure comparability across modalities and robustness against container-based variability.

Approximately 77 % of the dataset includes audio tracks, enabling consistent evaluation of cross modal coherence features. The videos encompass a diverse range of scenes, including studio environments, outdoor settings, domestic interiors, and multi speaker configurations. This heterogeneity reflects the operational variability encountered in telemedicine and social media content distribution.

Metadata for each clip were collected using a standardized JSON schema, including generator type, model family, post processing operations, codec structure, resolution, bitstream characteristics, and audiovisual synchronization indicators. The dataset spans multiple codec profiles, container brands, platform derived transformations, and degradation sequences. These include recompression, scaling, augmented reality filters, recapture from screens, and low bitrate transcoding. Such diversity ensures that the dataset captures real world distortions that frequently impair the performance of unimodal detectors.

The dataset was constructed exclusively from publicly accessible sources, consistent with recognized practices in open-source intelligence research. All personally identifiable information was removed or pseudonymized using salted hashing of publisher identifiers, removal of EXIF data, and elimination of geolocation cues. Processing was conducted under Article 6 and Article 89 of the General Data Protection Regulation, which permits the use of publicly available data for scientific research purposes when subject to appropriate safeguards. No private accounts or restricted content were accessed, and materials with potentially sensitive attributes were excluded from the repository.

These practices align with the standards of ethical data curation in multimedia forensics and the principles of fairness, accountability, and transparency recommended by contemporary guidelines for responsible AI research (2, 18).

Although the dataset was not originally restricted to medical contexts, its diversity and multimodal richness make it highly suitable for evaluating integrity verification systems relevant to clinical medicine and dentistry. Teleconsultations and remote diagnostic procedures frequently involve audiovisual recordings captured under uncontrolled environmental and technical conditions. These include variable lighting, heterogeneous room acoustics, fluctuating frame rates, and consumer grade hardware with limited stability. The broad distribution of resolutions, bitrates, face angles, motion dynamics, and acoustic profiles in the dataset approximates the real world variability of dental telemedicine scenarios.

Specific attributes of the dataset, such as the presence of speech driven facial reenactment, diverse prosodic patterns, and variable background noise, align closely with clinical tasks that depend on the assessment of mandibular motion, phonetic articulation, and extraoral facial symmetry. Consequently, the DeepFake RealWorld dataset provides an empirically grounded foundation for the design and evaluation of deepfake detection methods that must operate reliably within dental practice.

Classic benchmarks such as DFDC, FaceForensics plus plus, Celeb DF, and DeeperForensics offer valuable reference points but fall short of capturing the complexity of modern generative models and platform specific transformations. Most lack comprehensive audio tracks, provide limited variation in scene dynamics, and rely heavily on artifacts characteristic of earlier GAN based architectures. As a result, detectors trained on these datasets have been shown to exhibit limited cross-dataset generalization when evaluated under differing generative pipelines and distributional conditions (9, 17).

By incorporating diffusion based video synthesis, modern face reenactment, multimodal audio driven manipulation, and realistic distribution pipelines, the present dataset addresses these limitations. It provides a technologically and semantically representative environment necessary for evaluating multimodal coherence features that underpin the proposed detection framework. This level of realism is critical for clinical contexts, where diagnostic decisions depend on accurate and trustworthy audiovisual information.

## Methods

The multimodal deepfake detection framework was designed to ensure methodological transparency, reproducibility, and robustness under conditions of real world audiovisual degradation. The approach integrates visual, acoustic, and cross modal coherence features, with all thresholds derived exclusively from authentic recordings to support interpretability consistent with forensic and clinical standards while avoiding reliance on opaque supervised classifiers. All clips from the DeepFake RealWorld dataset were normalized to maintain comparability and stable measurement conditions. Video streams were transcoded to MP4 using the H.264 High profile at 30 frames per second, following ITU T H.264 and ISO/IEC 14496 10 specifications. Luminance and contrast normalization reduced variability introduced by platform dependent post processing. Audio tracks were resampled to 48 kHz, loudness normalized in accordance with EBU R128 and processed following ITU R BS.1770 4 recommendations (19, 20). Segmentation into fixed duration windows enabled consistent extraction of phonetic, prosodic, and room acoustic parameters, while bitstream level metadata such as sequence parameter sets, picture parameter sets, frame types, and group of pictures structure were retained for identifying encoding related anomalies.

**Figure 1:**
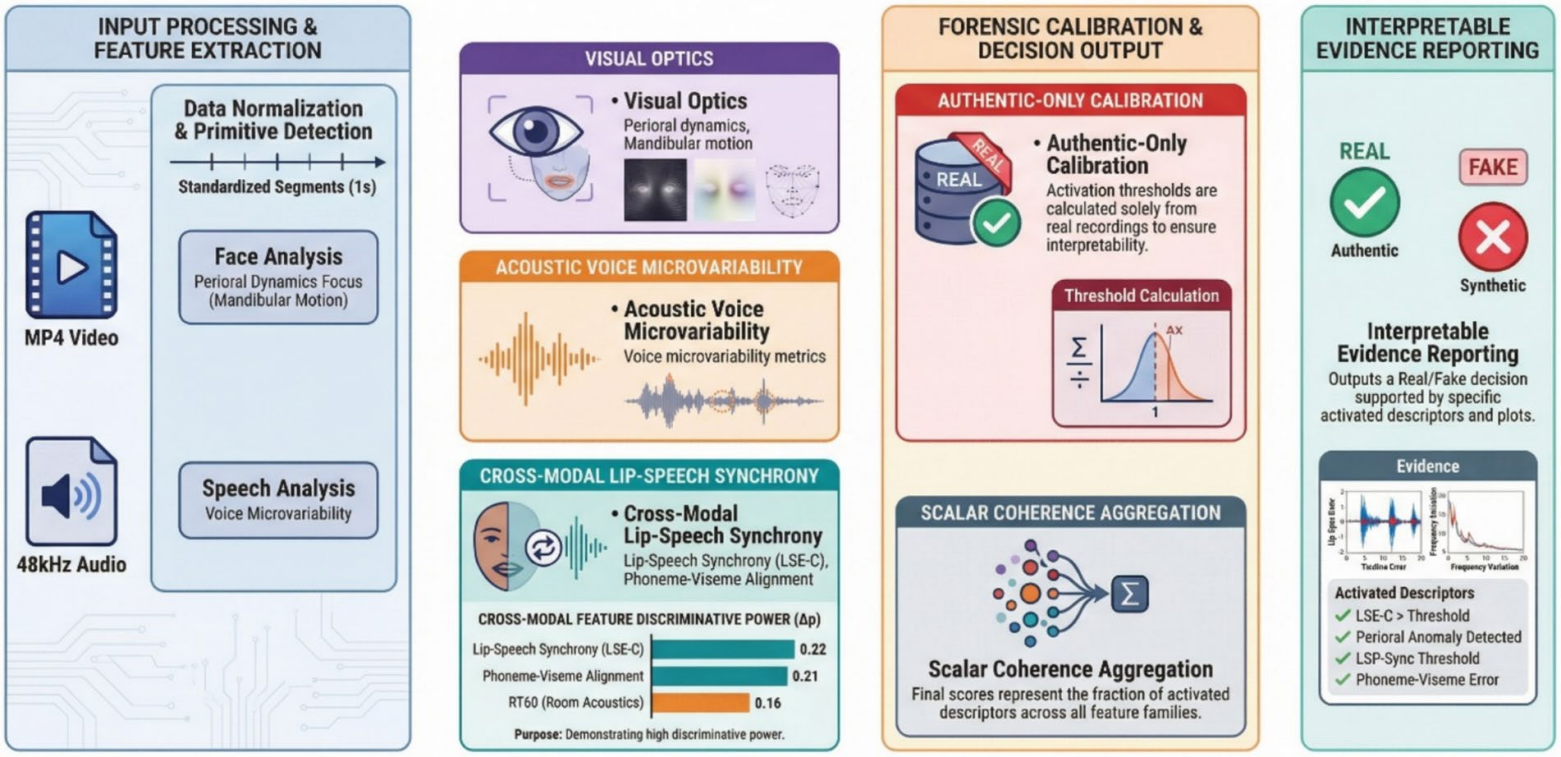
System architecture and workflow of the multimodal coherence based deepfake detector. The pipeline processes an input MP4 clip through normalization and segmentation, extracts visual, acoustic, and cross modal coherence descriptors, applies thresholds calibrated exclusively on authentic recordings to derive descriptor activations, aggregates activations into a scalar coherence score, and outputs the final fake or real decision together with an interpretable evidence report.

Figure 1 summarizes the complete workflow of the proposed detection framework from the input MP4 recording to the final fake or real decision. The diagram explicitly separates preprocessing, multimodal feature extraction, threshold calibration derived from authentic recordings, descriptor activation logic, and the final coherence score that yields the decision flag and an interpretable evidence report.

Visual features were selected to represent physically grounded markers of image formation, scene consistency, and biologically plausible facial dynamics (21). Radial distortion was estimated by fitting a camera model to facial landmarks, with anomalous curvature indicating either synthetic rendering or inconsistency with real optical systems. Rolling shutter deviation was assessed from the temporal behavior of vertical edges during motion, as synthetic content often lacks coherent sensor specific distortions. Vignetting was quantified through relative luminance falloff from center to periphery, capturing deviations from natural optical profiles (22). Texture based descriptors included discrete cosine transform coefficient histograms and entropy measures of quantization parameter sequences extracted directly from the bitstream, allowing detection of asymmetries and compression patterns uncharacteristic of natural camera encoders (2). Spatial coherence across skin, hair, and background regions was measured through orientation based texture entropy metrics. Dynamic visual cues were analyzed using RAFT optical flow to isolate non rigid micro motions of the ocular and perioral regions, which tend to be suppressed in synthetic reenactment (23). Mesh topology analysis of reconstructed three dimensional surfaces provided curvature based indicators of unnatural geometric deformations inconsistent with human facial anatomy.

Acoustic descriptors captured spontaneous variability in human speech production, room acoustics, and artifacts introduced by generative or compression pipelines. Fundamental frequency trajectories and perturbation measures such as jitter and shimmer were extracted as markers of natural vocal fold vibration, as synthetic voices typically show reduced micro variability due to vocoder smoothing. Mel frequency cepstral coefficient deviation from an authentic speech model was calculated using Mahalanobis distance to detect unnatural spectral modulation. Room acoustic properties were characterized by estimating reverberation time RT60 and the direct to reverberant energy ratio based on ISO 3382 1 methods (24), enabling assessment of whether the acoustic profile was plausible for the visual scene. Shorter RT60 in synthetic audio is typically consistent with a dry generation workflow in which the speech signal is produced in a close microphone, low reverberation condition and the room impulse response of the target environment is not explicitly rendered. In many practical pipelines, additional speech enhancement or dereverberation steps may further suppress late reverberation to improve intelligibility, which also reduces the estimated RT60. Therefore, the RT60 reduction observed in DeepFake RealWorld is interpreted here primarily as an indicator of dry generation or postprocessing rather than a limitation attributable to a specific vocoder architecture (25, 26). Ambient sound semantics were analyzed using large scale audio event classifiers including PANNs and YAMNet to identify inconsistencies between environmental audio cues and their corresponding visual context. Additional acoustic indicators included wavelet based detection of pre echo, aliasing, and transient smearing, as well as formant drift reflecting inconsistent trajectories of F1 to F3 relative to prosodic patterns.

Cross modal coherence features formed the core of the framework, capturing the alignment between facial motion, speech acoustics, identity, and environmental context. Lip speech synchronization metrics LSE D and LSE C quantified the temporal coupling between lip aperture dynamics and phonetic content, with synthetic or reenacted materials often exhibiting delayed or oversmoothed articulation. Phoneme viseme alignment was assessed by comparing phoneme boundaries predicted from acoustic analysis with viseme segments derived from facial landmarks, and misalignment was interpreted as evidence of cross modal desynchronization. Phoneme viseme alignment has been used in prior multimodal detection pipelines as a marker of audio visual inconsistency and lip articulation desynchronization (11, 12). In the present study, the distinguishing element is its clinical calibration for teledentistry. Viseme segmentation and its interpretation are focused on the perioral region and mandibular related dynamics that are clinically salient in remote dental assessment, including jaw opening and closing timing, the smoothness and plausibility of mandibular trajectory during speech, and asymmetries or oversmoothing that can confound interpretation of extraoral function and facial symmetry in dental consultations. Audiovisual delay was estimated through cross correlation between lip motion and amplitude envelopes, with deviations beyond forty five milliseconds exceeding ITU R BT.1359 3 tolerances for natural perception and reliably indicating manipulation (ITU-R (27)). Identity coherence was evaluated by computing cosine distance between facial embeddings from ArcFace and speaker embeddings produced by ECAPA TDNN, where divergences suggested dubbing or voice replacement (28, 29). Scene audio semantic agreement was measured by comparing visual semantic vectors from CLIP or PlacesCNN with audio event vectors from PANNs or YAMNet, allowing detection of implausible pairings such as indoor acoustics accompanying outdoor imagery. Prosody motion coupling between prosodic contours and head or jaw motion served as an indicator of natural speech related orofacial coordination, which is typically diminished in synthetic reenactment (30). Compared with the prior conference disclosure, the present manuscript includes additional coherence descriptors and reports their systematic evaluation across the full set of platform transformations considered in this paper (16).

To ensure interpretability and forensic reliability, feature selection relied on threshold based criteria computed solely from the distribution of authentic recordings. For right tailed features, thresholds were defined as the median plus two median absolute deviations, while left tailed features used corresponding percentiles. For interpretability, we distinguish descriptor violation from descriptor coherence. For each descriptor i, a violation indicator a_i equals 1 when the descriptor exceeds the authentic calibrated threshold in at least 10 % of frames within the analyzed window, and 0 otherwise. For reporting and visualization, we define the complementary coherence indicator c_i = 1 − a_i, so that c_i = 1 denotes conformity with authentic behavior. Discriminative strength was quantified using Δp as the difference in violation rates between synthetic and real clips, and PR as the ratio of violation rates in synthetic versus real content. Features were retained when p_real did not exceed 20 % and Δp was at least 0.15 or PR was at least 1.5, with correction for multiple comparisons using the Benjamini Hochberg procedure (31). The scalar coherence score S for a clip is computed as the mean of c_i across all descriptors.

**Figure 2:**
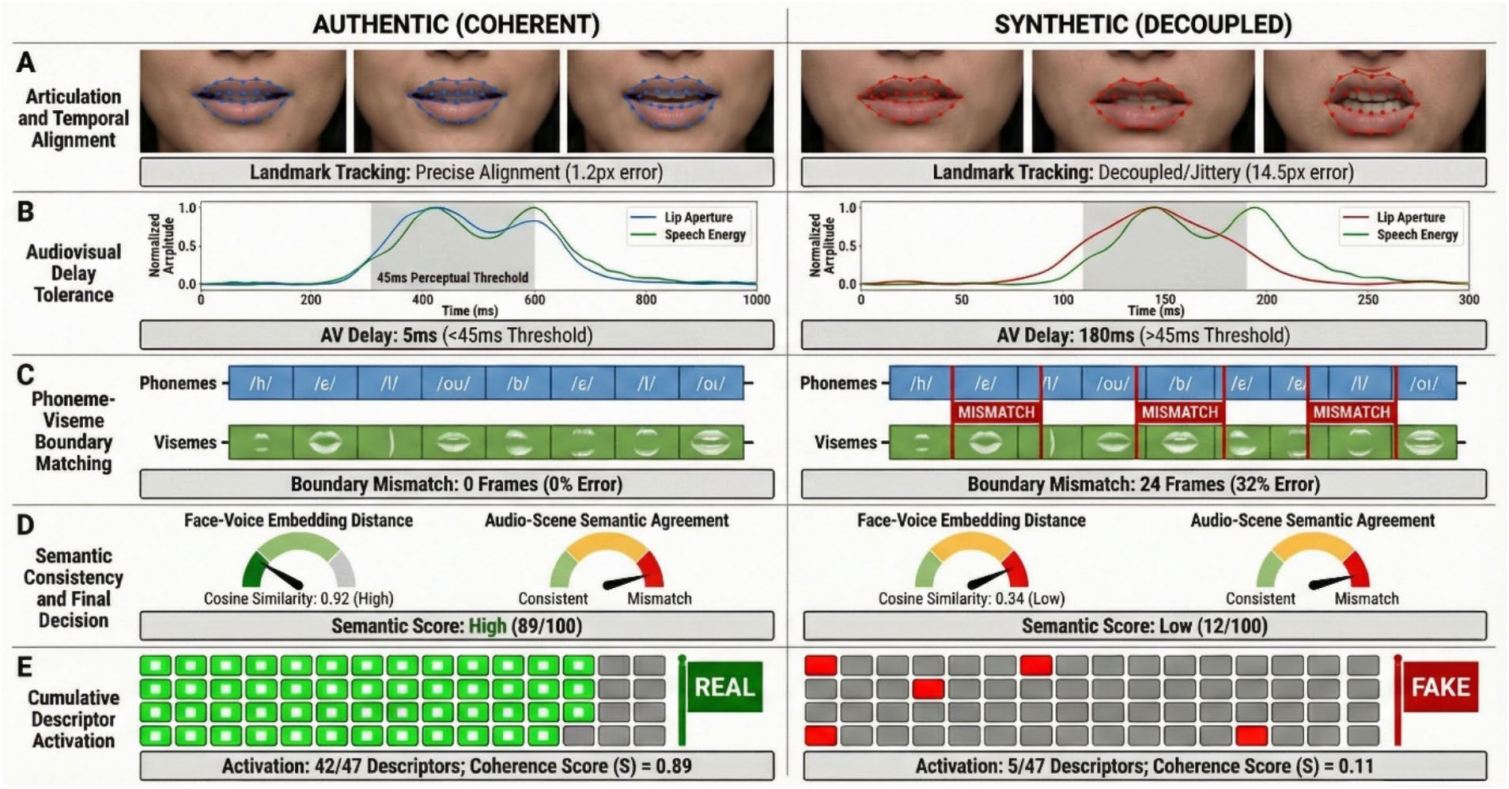
Representative qualitative evidence reports produced by the framework for authentic and synthetic samples, showing intermediate coherence signals and the final decision. **(A)** Perioral lip landmarks. **(B)** Audiovisual delay between lip aperture and speech energy. **(C)** Phoneme–viseme alignment. **(D)** Face–voice identity coherence and scene–audio semantic agreement; panel **(D)** reports cosine similarity corresponding to embedding cosine distance. **(E)** Descriptor coherence summary with coherence score S; panel **(E)** reports the number of coherent descriptors (∑ci) together with S. Values are from the displayed examples and are not dataset averages. Visual content is anonymized by cropping or blurring to prevent re-identification while preserving clinically relevant perioral dynamics.

The framework is designed to reduce false alarms caused by authentic anatomical variance or speech related idiosyncrasies. First, all thresholds are derived exclusively from authentic recordings and feature retention enforces a strict upper bound on violation rate in authentic content, which limits sensitivity to stable traits such as facial morphology, habitual articulation, accent, or benign speech disfluency. Second, we do not interpret any single descriptor, such as phoneme viseme mismatch, as sufficient evidence of manipulation. Instead, the decision is supported by concordant violations across multiple independent descriptor families, including cross modal synchrony, identity coherence, and scene audio semantic agreement, as summarized by the scalar coherence score computed as the fraction of coherent descriptors (c_i). Third, cases where only articulation related cues are violated, while identity coherence and acoustic scene plausibility remain consistent, are treated as low confidence and interpreted as potentially attributable to authentic speech impediments or anatomical conditions rather than synthetic generation. This design supports clinical use by prioritizing specificity and interpretability over aggressive detection. For each clip, the system produces an interpretable evidence report that lists violated descriptors, and visualizes the complementary coherence signals over time. This report format is used for the qualitative demonstrations in Figure 2 and is intended to facilitate clinical review and forensic accountability.

To provide an external point of reference, we benchmarked the proposed coherence framework against a standard open source deepfake detector based on the Xception convolutional architecture (32), as commonly used as a baseline in FaceForensics++ style evaluation (9). This baseline operates on face crops and is known to rely predominantly on local texture and compression related cues.

For the baseline, faces were detected and aligned for each clip, frames were sampled at a fixed rate, and each sampled frame was resized to the input resolution expected by the model. The baseline produced a per frame manipulation probability, which was aggregated into a clip level score by averaging probabilities across sampled frames. The same clip sets were used for evaluation as in the proposed framework, including the original clips and their transformed versions generated by recompression, resolution scaling, augmented reality filtering, and recapture operations.

To enable standard performance metrics for the proposed framework without introducing an opaque supervised classifier, we defined a scalar coherence score S for each clip as the mean of coherence indicators across the full descriptor set. Specifically, for each descriptor, we first compute a violation indicator a_i using thresholds derived exclusively from authentic recordings, and then define c_i = 1 − a_i. The coherence score is computed as S = (1/M) Σ_i c_i, where M is the number of descriptors. Performance was reported at the clip level using ROC AUC and balanced accuracy. Robustness was assessed by comparing results on the original clips and on the transformed clip sets under the same evaluation protocol.

Computational latency was quantified to evaluate the feasibility of real-time deployment in telemedicine applications. Measurements were conducted in a streaming-like configuration with a batch size of 1, processing contiguous 1-s audiovisual segments and excluding one-time model initialization overhead. For each method, we measured the processing time required for a 1-s segment and report the mean, standard deviation, and 95th percentile latency across N segments. Timings were obtained using a high-resolution monotonic timer and encompass all relevant steps including face detection and alignment, feature extraction, and final clip-level scoring, while excluding disk I/O operations. For the proposed coherence framework, descriptor extraction followed the same sampling and windowing parameters as those used in the main evaluation. For the Xception baseline, latency measurements included face cropping, frame resizing to the model’s expected input resolution, per-frame inference, and clip-level score aggregation. The experiments were conducted using the following hardware and software configuration: Intel Core i9-13900K CPU, NVIDIA RTX 4090 GPU, 128 GB RAM, Ubuntu 22.04 LTS, Python 3.10, and PyTorch 2.1 with CUDA 12.1. The final latency results are summarized in Table 1.

**Table 1:** Computational latency under a streaming configuration with batch size 1.

Method	Segment length	Mean latency	Std latency	P95 latency	Effective throughput
Proposed coherence framework	1 s	280 ms	45 ms	380 ms	2.63 segments/s
Xception baseline	1 s	120 ms	20 ms	160 ms	8.33 segments/s

Latency is reported per 1 s audiovisual segment as mean, standard deviation, and 95th percentile. Effective throughput is reported as segments processed per second under the same configuration.

Clinical relevance was evaluated through 60 simulated dental teleconsultations constructed by selecting clips from the DeepFake RealWorld dataset that meet clinically relevant criteria for remote dental assessment. No participants were recruited and no actors were recorded for this study. The individuals visible in authentic clips originate from publicly accessible recordings already present in the dataset, while manipulated counterparts represent synthetic or transformed recordings produced by the generative pipelines contained in DeepFake RealWorld. The simulation protocol focused on recordings with frontal facial views, sufficient visibility of the perioral region, active speech, and jaw related movements that are commonly observed during remote communication.

Each simulated teleconsultation was assessed using a predefined clinical checklist to define diagnostic compromise. The checklist included mandibular motion plausibility during speech, including jaw opening and closing timing, smoothness of mandibular trajectory, and left right symmetry, perioral dynamics, including lip closure adequacy and oral commissure micro movements, and phonetic clarity, defined as intelligibility and consistency of articulation for visually salient speech segments. In addition, plausibility of the acoustic scene was evaluated using room acoustic coherence, background noise consistency, and the absence of artifacts that could mask or distort speech assessment. A teleconsultation was considered diagnostically compromised when manipulation plausibly reduced interpretability of mandibular and perioral dynamics or speech clarity that would be required for extraoral functional screening in remote dentistry. This simulation based assessment provided an applied validation that links the proposed coherence descriptors to clinically meaningful failure modes in teledentistry.

## Results

**Figure 3:**
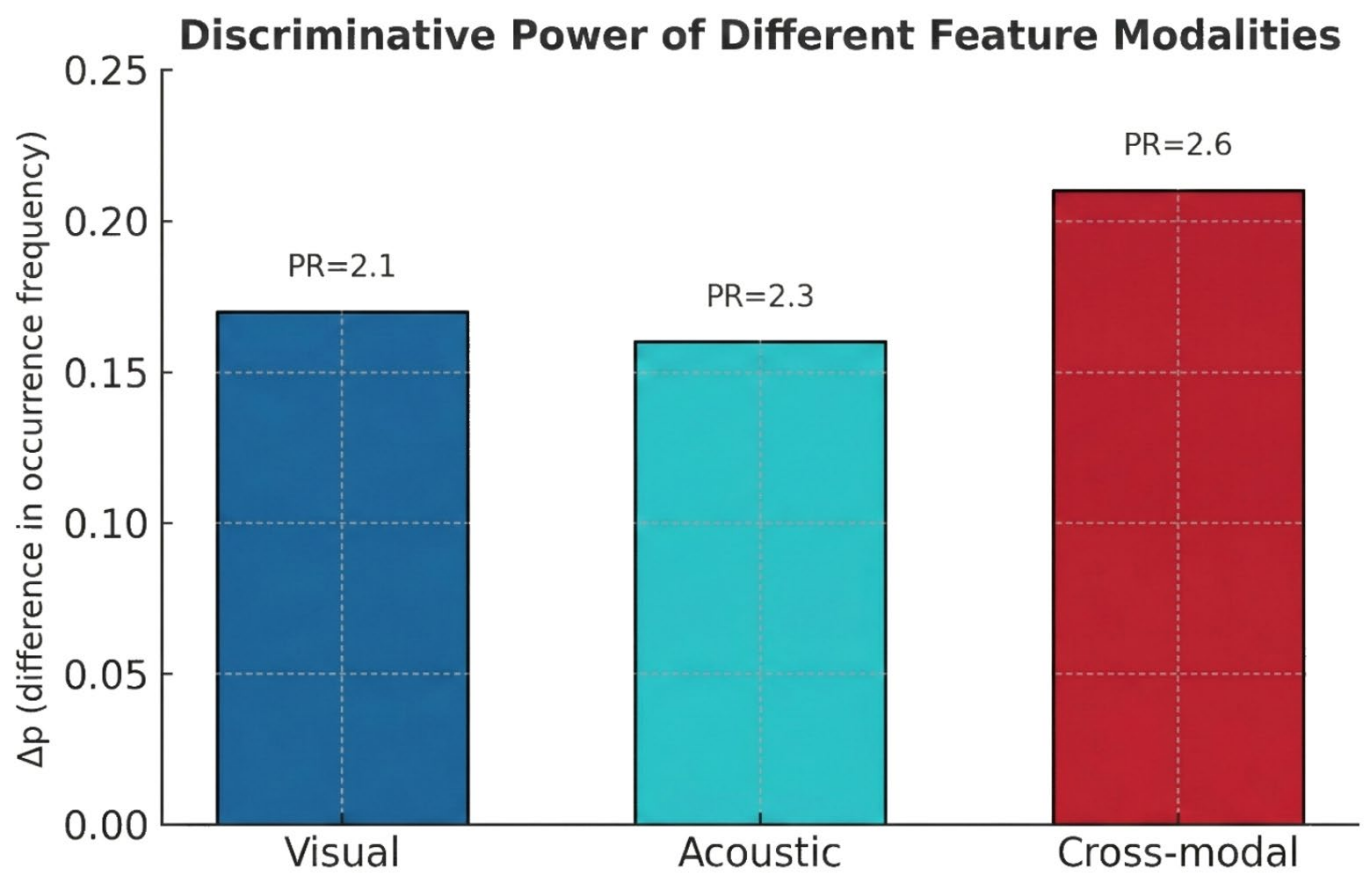
Discriminative power of visual, acoustic, and cross-modal features. Cross-modal metrics show the strongest separation (Δ*p* ≈ 0.21, PR ≈ 2.6) for robust, explainable deepfake detection in clinical use. Adapted from the authors’ own previously published work (16).

The multimodal framework was evaluated across 47 audiovisual descriptors, revealing consistent and interpretable separation between authentic and synthetic recordings. The results demonstrate that cross modal coherence features exhibit the highest discriminative power, while visual and acoustic features provide complementary stability across diverse degradation conditions (Figure 3). All thresholds were derived exclusively from authentic recordings, ensuring transparency and reproducibility of the evaluation process.

Cross modal descriptors yielded the strongest performance, with clear differences between real and synthetic clips based on temporal and semantic alignment. Lip speech synchronization metrics LSE D and LSE C showed the highest discriminative strength. Synthetic recordings exhibited elevated LSE D values and reduced LSE C values, producing *Δp* values between 0.21 and 0.22 and PR ratios ranging from approximately 2.5 to 2.7. Phoneme viseme mismatch further reinforced this separation, with desynchronization present in 37 % of synthetic videos compared with 14 % of real clips. We emphasize that phoneme viseme mismatch was interpreted as a manipulation indicator only when supported by concordant anomalies in independent coherence cues, reducing confounding by authentic articulation differences. Audiovisual delay estimates identified deviations exceeding 45 milliseconds in a significant proportion of manipulated content, consistent with the breakdown of natural speech articulation timing. Identity coherence demonstrated strong separation, with face-voice embedding similarity producing Δp equal to 0.19 and PR close to 2.5. Synthetic clips frequently showed inconsistencies between speaker characteristics and facial identity, particularly in cases involving audio replacement or reenacted dialog. Scene audio semantic consistency also reached high discriminative capacity, with Δp equal to 0.18 and PR equal to 2.6, indicating systematic mismatches between acoustic context and visual environment in manipulated recordings. Table 2 presents the quantitative summary of discriminative performance for visual, acoustic, and cross modal features.

**Table 2:** Discriminative performance of multimodal feature groups, including representative descriptors, Δp, prevalence ratio (PR), and their interpretability and clinical relevance.

Feature group	Representative features	Δp and PR	Interpretability and clinical relevance
Cross modal coherence	LSE D, LSE C, phoneme viseme mismatch, audiovisual delay, face-voice identity coherence, scene audio semantic agreement	Δp = 0.21–0.22, PR = 2.5–2.7; identity Δp = 0.19, PR = 2.5; scene audio Δp = 0.18, PR = 2.6	Strongest discriminators. Reflect physiological synchrony of speech and orofacial motion; essential for assessing articulation and mandibular movement in teleconsultations.
Acoustic features	F0 variability, jitter, shimmer, RT60, DRR, MFCC deviation, formant drift	F0 variability −22 percent; jitter −31 percent; shimmer −27 percent; RT60: 0.12 s vs. 0.28 s (Δp = 0.16, PR = 2.3)	Capture natural microvariability of human speech and room acoustics; relevant for judging clarity of speech and verifying plausibility of clinical environments.
Visual dynamic features	Facial micro motion, mesh topology stability	Micro motion Δp = 0.19	Reflect real biomechanical dynamics of perioral and ocular regions; useful for evaluating extraoral symmetry.
Visual geometric and photometric features	Radial distortion, rolling shutter deviation, vignetting	Radial distortion Δp = 0.17, PR = 2.1; rolling shutter Δp = 0.16, PR = 2.0	Derived from physical camera models; reliable in low bitrate conditions; valuable for forensic transparency.
Bitstream and texture descriptors	DCT histogram asymmetry, QP entropy, GOP irregularity	Moderate discriminative power; PR ≈ 1.5	Identify generator-specific artifacts and compression traces not consistent with real camera pipelines.

Cross modal coherence features demonstrate the strongest separation between authentic and synthetic recordings, while acoustic and visual features provide complementary indicators of natural speech dynamics, biomechanical facial motion, optical consistency, and environmental plausibility relevant to dental teleconsultations.

Acoustic descriptors captured deviations in natural voice production, environmental acoustics, and processing artifacts. Synthetic recordings displayed reduced natural variability of the speech signal, reflected in lower fundamental frequency dynamics, diminished jitter and shimmer, and spectral smoothing characteristic of neural vocoders. Average reductions reached approximately 22 % for F0 variability, 31 % for jitter, and 27 % for shimmer. Reverberation time RT60 was markedly shorter in synthetic recordings, with a median value of 0.12 s compared with 0.28 s for authentic clips. This pattern is most consistent with synthetic speech being generated or edited in a comparatively dry condition and then inserted into the video without an explicit acoustic rendering step that would match the target scene through convolution with a room impulse response. When late reverberation is absent or suppressed by enhancement or dereverberation, the decay tail is shortened, yielding lower RT60 and typically higher direct to reverberant energy ratios (25, 26). This produced a Δp value of 0.16 and PR equal to 2.3. These results confirm that synthetic audio generation frequently fails to reproduce realistic room acoustics or the natural micro variability of human phonation, even when integrated with visually coherent reenactment.

To facilitate reproducibility and clinical interpretability despite restricted access to raw data, Figure 2 provides representative visual demonstrations of the framework output on authentic and synthetic samples. For each sample, we show the intermediate coherence signals and the final evidence summary, including lip motion versus speech energy alignment, phoneme viseme boundary consistency, identity coherence, scene audio semantic agreement, and the resulting descriptor coherence summary (c_i) used to compute the coherence score S. Representative cases were selected to reflect typical teleconsultation conditions with visible perioral dynamics and active speech, and are reported with anonymized identifiers and metadata.

Ambient acoustic semantics provided additional discriminatory strength, as mismatches between environmental sound cues and the visual scene were more common in manipulated videos. Formant drift analysis further revealed inconsistent relationships between formant trajectories and prosodic contours in synthetic speech, consistent with independently generated or weakly coupled modalities.

Visual descriptors contributed robust complementary information, particularly for physically grounded cues related to camera optics, facial dynamics, and compression characteristics. Radial distortion estimates produced Δp equal to 0.17 and PR equal to 2.1, reflecting the absence or instability of real optical signatures in synthetic content. Rolling shutter deviation similarly revealed inconsistencies, achieving Δp equal to 0.16 and PR equal to 2.0. Both measures proved resilient across degradation conditions, as synthetic generation pipelines typically do not replicate sensor specific temporal distortions. Micro motion analysis demonstrated reduced high frequency non rigid facial dynamics in synthetic content, producing Δp equal to 0.19. Mesh topology metrics identified geometric inconsistencies in reconstructed three dimensional facial structures, although with lower discriminative power than cross modal features. Bitstream based metrics captured anomalies in quantization parameter entropy, group of pictures structure stability, and SPS PPS patterns. While individually weaker than the dynamic and cross modal descriptors, these features helped identify synthetic clips with generation specific encoding artifacts.

The framework exhibited consistent robustness under common platform specific degradations. Across the tested range of transformations, including H.264 to H.265 recompression, resolution scaling from 480p to 1440p, augmented reality filters, and recapture operations, the majority of features maintained a performance loss below 15 %.

To address external benchmarking, we compared the proposed coherence score against the Xception baseline on the DeepFake RealWorld dataset. Table 3 reports ROC AUC and balanced accuracy for both methods on the original clips and on the principal transformed subsets used in the robustness analysis. The coherence based approach preserves discriminability under platform transformations, whereas the texture oriented baseline shows larger performance declines, supporting the claim that coherence features are more stable under real world distribution shifts.

**Table 3:** Benchmark comparison against an open source Xception baseline on DeepFake RealWorld.

Condition/transformation	Proposed coherence ROC AUC	Proposed coherence balanced accuracy	Proposed ΔROC AUC vs original	Xception ROC AUC	Xception balanced accuracy	Xception ΔROC AUC vs original
Original clips (no transformation)	0.94	0.89	0.00	0.96	0.92	0.00
H.264 recompression (medium bitrate)	0.91	0.86	0.03	0.82	0.78	0.14
Resolution scaling (down to 480p)	0.90	0.85	0.04	0.79	0.74	0.17
Augmented reality filters	0.89	0.84	0.05	0.77	0.72	0.19
Recapture (screen recording)	0.88	0.83	0.06	0.74	0.69	0.22
Mean across transformations	0.90	0.85	0.04	0.78	0.73	0.18

Clip level ROC AUC and balanced accuracy are reported on original clips and on clips subjected to platform transformations used in the robustness analysis. Average across transformations denotes the arithmetic mean across the transformed conditions only and excludes the original clips.

To confirm practical viability in telemedicine, we quantified computational latency for streaming inference of both the proposed framework and the Xception baseline (Table 1).

Cross modal descriptors were the most resilient, reflecting the difficulty of preserving physiologically accurate audiovisual synchrony under synthetic manipulation, even after heavy compression. Acoustic features showed moderate sensitivity to low bitrate encoding but retained discriminative capacity under all tested conditions. Visual descriptors dependent on fine grained texture exhibited expected reductions in discriminability under aggressive compression, whereas geometric and temporal features remained stable.

Sixty simulated dental teleconsultations were analyzed to assess clinical relevance. The simulated teleconsultations were not based on newly recorded participants but on a predefined subset of dataset clips selected to reflect typical remote dentistry recording conditions. Diagnostic compromise was defined using the clinical checklist described in Methods, with emphasis on mandibular motion plausibility, perioral dynamics, and phonetic clarity as the primary indicators that could affect extraoral functional interpretation. Manipulated recordings consistently produced elevated Δp values with a mean of 0.18 ± 0.04 and PR equal to 2.3 ± 0.2 across key multimodal descriptors. The most common anomalies included lip motion desynchronization, inconsistent mandibular movement during speech, divergence between acoustic environment and scene context, and unnatural attenuation of vocal micro variability.

These inconsistencies directly affected the interpretability of clinically relevant cues such as occlusal function, articulation clarity, and symmetry of extraoral facial dynamics. In several cases, manipulated recordings could plausibly alter clinical judgment or obscure diagnostic indicators. The results confirm the practical applicability of multimodal coherence metrics for supporting medico legal verification and integrity assessment in telemedicine.

## Discussion

The results demonstrate that multimodal coherence offers a robust and interpretable foundation for detecting synthetic audiovisual content under realistic conditions of acquisition, compression, and distribution. Compared with unimodal detectors that rely on texture inconsistencies or spectral artifacts, the proposed approach captures deviations that arise from disrupted physiological, acoustic, and semantic relationships between modalities. This multimodal perspective is particularly valuable in domains such as clinical dentistry, where diagnostic accuracy depends on coherent interaction between facial motion, speech, and contextual audiovisual cues.

Cross modal metrics consistently achieved the strongest discriminative performance, confirming prior observations that temporal alignment between visual and acoustic modalities is difficult to synthesize with high fidelity. Modern generative models have substantially reduced local artifacts characteristic of earlier GAN based architectures, yet they continue to struggle with accurate modeling of lip motion synchrony, phoneme viseme alignment, and physiological prosody motion coupling (1, 2, 11, 12, 17). In this manuscript, phoneme viseme alignment is not claimed as a novel cue. The added contribution is its teledentistry oriented calibration and interpretation in conjunction with additional coherence descriptors that emphasize mandibular and perioral dynamics relevant to dental diagnostics. The elevated Δp and PR ratios observed for LSE D, LSE C, and phoneme viseme mismatch reinforce the value of these features as reliable, degradation resistant indicators of manipulation.

Identity coherence further contributed strong discriminative capability. The inability of synthetic pipelines to maintain consistent speaker identity across both face and voice indicates that generative audio and visual subsystems often operate with limited mutual conditioning. This aligns with evidence that face-voice identity consistency remains an open challenge in multimodal generation research. Similarly, the observed mismatches between ambient acoustic context and visual scene semantics highlight the generator’s limited capacity to encode or reconstruct plausible environmental relationships.

An important outcome of this study is the demonstrated resilience of multimodal coherence features to platform dependent transformations. Real world teleconsultations and user generated content frequently undergo multi stage compression, resolution changes, color space transformations, and network induced distortions. Traditional detectors have been shown to exhibit reduced robustness under distributional shifts when trained on narrowly constructed benchmark datasets (1, 9, 17). In contrast, the proposed framework maintained performance declines below 15 % for the majority of features, even under aggressive recompression or recapture. This observation is consistent with the external benchmark in Table 3, where the Xception baseline exhibits larger declines under the same transformation conditions.

This stability is attributable to the physiological and semantic grounding of cross modal descriptors. While compression can obscure high frequency visual patterns or attenuate subtle audio fluctuations, it rarely introduces or corrects temporal misalignments between speech and facial articulation. As a result, coherence driven metrics remain detectable even after heavy degradation. These characteristics make the approach suitable for clinical applications where recordings originate from heterogeneous devices and variable network conditions.

The present findings have significant implications for clinical dentistry and medicine. Video based assessment is increasingly integrated into remote consultations, postoperative monitoring, and patient education. Deepfakes that disrupt cross modal coherence can distort these cues, potentially affecting diagnostic outcomes or medico legal documentation.

Simulated teleconsultations revealed that manipulated recordings produce inconsistencies in mandibular motion trajectories, lip synchrony, and prosodic facial coupling. For example, uncoordinated jaw motion during the articulation of bilabial consonants or reduced micro movement amplitude near the oral commissures can mimic or mask clinical signs relevant to temporomandibular disorders. Inaccurate ambient acoustics or unnatural reverberation profiles may affect interpretation of speech clarity or mask auditory manifestations of airway obstruction. In particular, the shorter RT60 observed in synthetic samples is more plausibly explained by omission of room acoustic rendering and by dereverberation oriented enhancement in generation or editing workflows, rather than by a single identifiable vocoder limitation (25, 26). These distortions underscore the importance of authenticity verification in remote medical assessment (2, 5).

The proposed framework provides clinicians with a transparent method for assessing audiovisual integrity based on measurable physiological and acoustic patterns rather than opaque machine learning predictions. This supports the alignment of telemedicine workflows with emerging standards of explainability and accountability in medical AI.

A key advantage of the presented approach is its reliance on threshold based analysis derived exclusively from authentic data distributions. This design avoids the black box behavior of end to end supervised detectors and ensures that each feature corresponds to a physically or biologically meaningful phenomenon. Such interpretability is essential for forensic applications, legal proceedings, and compliance with explainability frameworks that increasingly govern the use of AI in medical and security contexts (18, 33).

Furthermore, the use of independent, real only thresholding mitigates the risk of model overfitting to generation specific artifacts, a common limitation of discriminative deep learning models. By ensuring that p_real remains below a strict upper bound, the method enforces a transparent calibration strategy that clearly delineates the confidence of decisions and reduces false alarms in clinical settings.

To contextualize the proposed approach against a standard learning based detector, we benchmarked against an open source Xception baseline (9, 32). On DeepFake RealWorld, the baseline shows larger performance declines under the platform transformations evaluated here, whereas the coherence score preserves discriminability, as summarized in Table 3. The multimodal coherence approach addresses these shortcomings by combining domain grounded descriptors across vision, acoustics, and semantics, achieving performance stability not observed in purely visual or spectral methods. Importantly, multimodal coherence is complementary rather than competitive with learning based approaches. The descriptors identified in this study can serve as interpretable priors, calibration anchors, or attention regulators in future hybrid models that integrate coherence based constraints with high capacity neural networks. This hybrid direction aligns with emerging trends in explainable media forensics and trustworthy medical AI (2, 33, 34).

Future work should incorporate multimodal data from clinical workflows, including frontal face recordings synchronized with intraoral scans. Additionally, the increasing prevalence of three dimensional face synthesis and volumetric video generation introduces new challenges for coherence analysis, particularly for rotational and depth dependent features.

Further research is warranted on the calibration of multimodal coherence metrics across diverse languages, accents, and speech disorders that may influence articulation patterns and phoneme viseme alignment in clinical populations. In the current framework, isolated articulation related deviations are not treated as sufficient evidence of manipulation and are interpreted in conjunction with independent coherence cues such as identity consistency and scene audio plausibility, which reduces confounding by authentic anatomical variance or speech impediments. Finally, integration of the framework into real time telemedicine systems requires optimization for low latency processing and secure transmission protocols. Our latency measurements in a streaming like setting show a P95 processing time of 380 ms per 1 s audiovisual segment for the proposed coherence framework, supporting feasibility for near real time integrity verification when integrated as a background verification step.

To ensure transparency, we explicitly cite the prior conference communication and delineate the additional contributions of this manuscript, namely the full reproducible method specification, the teledentistry focused simulation, refined thresholds, and the newly added external benchmark results.

## Conclusion

This study demonstrates that multimodal coherence provides a reliable and interpretable foundation for deepfake detection in clinical dentistry and related medical applications. By integrating visual, acoustic, and cross modal descriptors that reflect physically, physiologically, and semantically grounded relationships, the framework captures anomalies that persist even when synthetic content undergoes substantial platform specific degradation. The analysis conducted on the DeepFake RealWorld dataset confirms that features based on lip speech synchrony, phoneme viseme coordination, audiovisual latency, identity coherence, and scene audio semantic consistency produce consistent separation between real and manipulated content, with Δp values around 0.20 and PR ratios near 2.5.

Acoustic descriptors, including measures of vocal micro variability and room acoustics, and visual features related to optical geometry and sensor behavior, provide complementary discriminatory power. Their combined performance ensures that the system remains robust under compression, scaling, and post processing conditions common in teleconsultations. This robustness makes the approach particularly well suited for remote dental diagnostics, where recordings originate from heterogeneous devices and uncontrolled acoustic environments.

The explainable design of the framework, based on thresholds derived exclusively from authentic data, supports transparency and forensic accountability. Each metric corresponds to a specific, interpretable audiovisual phenomenon, enabling clinicians and forensic analysts to understand and justify the detection outcome. This aligns with emerging standards for trustworthy and medically acceptable artificial intelligence systems.

The clinical evaluation using simulated dental teleconsultations highlights the practical importance of verifying audiovisual integrity in remote care. Deepfake manipulations can distort key diagnostic cues, including mandibular motion, articulation clarity, extraoral symmetry, and ambient acoustic context. By revealing such inconsistencies, the proposed method enhances medico legal reliability and strengthens the credibility of telemedicine workflows. Incorporating coherence constraints into hybrid learning based detectors represents a promising direction for scaling the approach to large clinical datasets and real time applications.

Overall, the results establish multimodal coherence as a robust, clinically relevant, and scientifically transparent standard for deepfake detection, offering a strong foundation for future work in audiovisual integrity verification in digital health.

## Data Availability

The raw data supporting the conclusions of this article will be made available by the authors, without undue reservation.

## References

[1] Pei G Zhang J Hu M Zhang Z Wang C Wu Y . Deepfake generation and detection: a benchmark and survey. *arXiv* (2024). [Epub ahead of preprint].

[2] Verdoliva L. Media forensics and deepfakes: an overview. IEEE J Sel Top Signal Process. (2020) 14:910–32. doi: 10.1109/jstsp.2020.3002101

[3] El-Tallawy SN Pergolizzi JV Vasiliu-Feltes I Ahmed RS LeQuang JAK Alzahrani T . Innovative applications of telemedicine and other digital health solutions in pain management: a literature review. Pain Ther. (2024) 13:791–812. doi: 10.1007/s40122-024-00620-7, 38869690 PMC11255158

[4] Hsu CC Tsai MY Yu CM. Securing healthcare data integrity: deepfake detection using autonomous AI approaches. IEEE J Biomed Health Inform. (2025) 10, 1460–1473. doi: 10.1109/JBHI.2025.3586963 40633045

[5] Kaur A Hoshyar AN Wang X Xia F (2024). Beyond deception: exploiting deepfake technology for ethical innovation in healthcare.

[6] Berko SN. Securing the digital health ecosystem: a framework for safeguarding patient data in telemedicine. Int J Innov Sci Res Technol. (2025) 10:1460–1473. doi: 10.38124/ijisrt/25oct811

[7] Susilawati Sari HE Nurhaeni H Hardini M Agustian H Leffia A . Digital health innovations and telemedicine effectiveness in remote patient monitoring. Proc Int Conf Commun Inf Technol. (2025).

[8] Hashmi A Shahzad SA Lin CW Tsao Y Wang HM. Understanding audiovisual deepfake detection: techniques, challenges, human factors and perceptual insights. *arXiv* (2024). [Epub ahead of preprint].

[9] Rossler A Cozzolino D Verdoliva L Riess C Thies J Nießner M. FaceForensics++: learning to detect manipulated facial images. Proceedings of the IEEE International Conference on Computer Vision. (2019) 1–11.

[10] Raza MA Malik K (2023). Multimodaltrace: deepfake detection using audiovisual representation learning.

[11] Datta SK Ranga T Sun C Lyu S (2025). PIA: Deepfake detection using phoneme-temporal and identity-dynamic analysis. Proceedings of the IEEE International Conference on Computer Vision: 1596–1606.

[12] Shahzad SA Hashmi A Peng YT Tsao Y Wang HM (2023). AV-lip-sync+: leveraging AV-HuBERT to exploit multimodal inconsistency for video deepfake detection *arXiv* [Epub ahead of preprint].

[13] Du Y Wang Z. Luo Y Piao C Yan Z Li H . (2025). CAD: a general multimodal framework for video deepfake detection via cross-modal alignment and distillation. *arXiv* [Epub ahead of preprint].

[14] Inchara A Chakrapani S Rathod A Sehreen A Dhanalakshmi S. Multimodal deepfake detection frameworks: a survey. Int J Sci Res Eng Manag. (2025) 9:1–9. doi: 10.55041/IJSREM52704

[15] Tariq S Woo SS Singh P Irmalasari I Gupta S Gupta D. (2025). From prediction to explanation: multimodal, explainable, and interactive deepfake detection framework for non-expert users. *arXiv* [Epub ahead of preprint].

[16] Jędrasiak K Bijoch J (2025). Integration of multimodal coherence features for robust and explainable deepfake detection with clinical dentistry use cases. Proc 46th Int bus Inf Manag Assoc Conf (IBIMA).

[17] Dolhansky B Bitton J Pflaum B Lu J Howes R Wang M (2020). The Deepfake detection challenge (DFDC) dataset. *arXiv* [Epub ahead of preprint].

[18] Tucci C Della Greca A Tortora G Francese R. Explainable biometrics: a systematic literature review. J Ambient Intell Human Comput. (2024). doi: 10.1007/s12652-024-04856-1

[19] EBU. Recommendation R128: Loudness normalisation and permitted maximum level of audio signals. Grand-Saconnex: European Broadcasting Union (2011).

[20] ITU-R. BS.1770–4: Algorithms to measure audio programme loudness and true-peak audio level. Geneva: International Telecommunication Union (2015).

[21] Wang J Wu B Liu L Liu Q. FauForensics: boosting audio-visual deepfake detection with facial action units. *arXiv* [Epub ahead of preprint]. (2025).

[22] Ray S. Applied photographic optics. London: Routledge (2002).

[23] Teed Z Deng J (2020). RAFT: recurrent all-pairs field transforms for optical flow. European Conference on Computer Vision:402–419.

[24] ISO. 3382–1: Acoustics – Measurement of room acoustic parameters - part 1: Performance spaces. Geneva: International Organization for Standardization (2009).

[25] Chowdhury S Ghosh S Dasgupta S Ratnarajah A Tyagi U Manocha D (2023). Adverb: visually guided audio dereverberation. Proceedings of the IEEE International Conference on Computer Vision:7884–7896.

[26] Su J Jin Z Finkelstein A (2021). HiFi-GAN-2: studio-quality speech enhancement via generative adversarial networks conditioned on acoustic features. Proc IEEE workshop Appl signal process audio Acoust (WASPAA):166–170.

[27] ITU-R. BT.1359–1: Relative timing of sound and vision for broadcasting. Geneva: International Telecommunication Union (1998).

[28] Deng J Guo J Xue N Zafeiriou S (2019). ArcFace: additive angular margin loss for deep face recognition. Proceedings of the IEEE conference on computer vision and pattern Recognition:4690–4699. 10.1109/TPAMI.2021.3087709 34106845

[29] Desplanques B Thienpondt J Demuynck K (2020). ECAPA-TDNN: emphasized channel attention, propagation and aggregation in TDNN-based speaker verification. *arXiv* [Epub ahead of preprint].

[30] Yoon S Byun S Jung K (2018). Multimodal speech emotion recognition using audio and text. IEEE spoken language technology workshop:112–118.

[31] Benjamini Y Hochberg Y. Controlling the false discovery rate: a practical and powerful approach to multiple testing. J R Stat Soc Series B Stat Methodol. (1995) 57:289–300. doi: 10.1111/j.2517-6161.1995.tb02031.x

[32] Chollet F (2017). Xception: deep learning with depthwise separable convolutions. Proceedings of the IEEE conference on computer vision and pattern Recognition. 1251–1258.

[33] Doshi-Velez F Kim B (2017). Towards a rigorous science of interpretable machine learning *arXiv* [Epub ahead of preprint].

[34] Durall R Keuper M Keuper J (2020). Watch your up-convolution: CNN-based generative deep neural networks are failing to reproduce spectral distributions. Proceedings of the IEEE conference on computer vision and pattern Recognition: 7890–7899.

